# Advances and Opportunities in Passive Wake-Up Radios with Wireless Energy Harvesting for the Internet of Things Applications

**DOI:** 10.3390/s19143078

**Published:** 2019-07-12

**Authors:** Hilal Bello, Zeng Xiaoping, Rosdiadee Nordin, Jian Xin

**Affiliations:** 1 College of Communication Engineering, Chongqing University, Chongqing 400044, China; 2 Centre of Advanced Electronic & Communication Engineering, Faculty of Engineering and Built Environment, Universiti Kebangsaan Malaysia, UKM Bangi, Selangor 43600, Malaysia

**Keywords:** Energy efficiency, energy harvesting, green communication, Internet of Things, MAC protocols, wake-up radio, wireless sensor network

## Abstract

Wake-up radio is a promising approach to mitigate the problem of idle listening, which incurs additional power consumption for the Internet of Things (IoT) wireless transmission. Radio frequency (RF) energy harvesting technique allows the wake-up radio to remain in a deep sleep and only become active after receiving an external RF signal to ‘wake-up’ the radio, thus eliminating necessary hardware and signal processing to perform idle listening, resulting in higher energy efficiency. This review paper focuses on cross-layer; physical and media access control (PHY and MAC) approaches on passive wake-up radio based on the previous works from the literature. First, an explanation of the circuit design and system architecture of the passive wake-up radios is presented. Afterward, the previous works on RF energy harvesting techniques and the existing passive wake-up radio hardware architectures available in the literature are surveyed and classified. An evaluation of the various MAC protocols utilized for the novel passive wake-up radio technologies is presented. Finally, the paper highlights the potential research opportunities and practical challenges related to the practical implementation of wake-up technology for future IoT applications.

## 1. Introduction

IoT technology is expected to offer a smart solution to users. However, the sad reality is the battery technology is still far away from being smart [1]. Often, users encountered shorter device lifespan. The major problem faced by IoT devices is the limited energy source. Once the device battery is drained [1,2], communication outages will occur before battery replacement, which potentially extends to other nodes since they rely on the dead node to relay the data. Hence, the availability of the power in the sensor nodes is very important to allow reliable communication. This makes energy efficiency and power management crucial in future IoT applications.

The transceiver (communication radio) accounts for the highest amount of energy consumption in the sensor node [3,4]. This is caused by a phenomenon known as idle listening. Idle listening occurs when the node has to stay active to the communication medium and listen for the incoming signal from neighbors, even when it is not actively sending or receiving any information. However, idle listening is necessary to avoid data latency and packet retransmission when the destination node is in a deep sleep during transmission. Another problem causing power wastage in the node’s transceiver is overhearing. It happens while listening for incoming signals, whereby the sensor node may receive irrelevant signals [5].

### 1.1. Solutions for Idle Listening

The following solutions have been proposed and used to address the challenges of idle listening and overhearing.

#### 1.1.1. Duty Cycling

Duty cycling, also known as ‘sleep schedule’, has been a crucial design consideration to prolong the lifetime of a wireless sensor network (WSN) [6,7]. It mitigates the energy consumption due to idle listening and overhearing in the sensor nodes. The radio is kept in a sleep mode and is switched to active mode from time to time via its internal synchronization clock to transmit or receive data. The major challenge of the duty cycling is to wake-up the nodes at the exact time when packets are sent to it, i.e., being received. If the sleep period is scheduled to be longer than the active period, more energy is saved, but there is an increased probability of packet missing during sleep time and network latency due to the increased waiting period. There is also increased overhead due to synchronization packets used within the nodes to schedule the wake-up time in duty cycling, hence increasing the energy wastage. Several techniques [8] have been proposed to address this challenge such as the use of spatial scheduling [9] or cognitive radio [10].

#### 1.1.2. Active and Passive Wake-Up Radio

Another solution to idle listening and overhearing in a network system is by using the wake-up radio, which a low power radio is attached to the main radio in charge of waking it up when an incoming signal is sensed. Having this device, the main radio can stay in sleep mode all the time without having to bother waking up and listening for incoming signals from time to time. This completely solves the problem of idle listening in the sensor node with no time synchronization overhead. The wake-up message and the data can be communicated in different channels, which makes it possible for the two message types to be exchanged simultaneously, hence reduce the possibility of collision. Several hardware designs and protocols have been developed to improve the performance and efficiency of the wake-up radios. Authors have extensively reviewed and analyzed the concept on many occasions [11,12].

There are two types of wake-up radios based on the power usage, namely; active and passive wake-up radios. The active wake-up radio is a second radio with low power that receives a continuous, external power supply such as batteries. Compared with active wake-up radios, passive wake-up radios do not require energy from the battery or any other power supply source connected physically. However, they harvest energy from the transmitted wake-up signal. Although passive wake-up radios require minimal energy through this process, the receiver sensitivity for passive wake-up radios is relatively lower, hence the wake-up range becomes shorter.

There is also ultralow power wake-up solution, where few components (such as the microcontroller, comparator, etc.) in the wake-up radio circuitry are battery powered, while the remaining components are powered with the energy from ambient energy or incoming signal. These designs [13,14,15] operate within the nanowatt power range and reduce power consumption but operate within a limited communication range. Passive wake-up radios, however, require no battery to operate, as their circuit is entirely powered passively from the ambient energy or incoming RF signal.

### 1.2. Related Works

Unlike the active wake-up radios, which has been in the limelight for the past few years and has been reviewed substantially, passive wake-up has not been largely reviewed. General hardware survey of wake-up radios is presented in [16,17], whereby their hardware layer characteristics are studied. Similar studies consider the advantages of wake-up radios in reducing network latency and increasing reliability [18,19] while others highlight their benefits for improving the energy efficiency of individual nodes [17,20,21]. Few studies [22,23] considers the passive (energy harvesting) wake-up radio hardware. Another study [24] presented a review on the state-of-the-art in energy harvesting from ambient energy for environmental monitoring applications while [25] having passive hardware design as a section of their study. The authors in [26] presented a study on power management techniques for wake-up radios.

On the other hand, many other authors have reviewed the MAC protocols for the wake-up radios [27,28,29,30], with only a few considering its combination with the energy harvesting system [31,32,33]. Some surveys consider the receiver only [17,34], while others [35], made a comparison of some of the wake-up radio schemes. Another study [36] explains the benefits, challenges, and applications of the wake-up radio while surveying some of the available MAC protocols used. The analytical models for the energy efficiency of wake-up radio are described in [37], while the mathematical models for the power consumption are derived in [38]. The work in [39] analyzed three wake-up radio MAC protocols and [40] analyzed the duty cycled MAC protocols. Some other studies highlighted the routing protocols [41,42,43,44] in WSN independently and in the multilayer application in [45,46].

At present, there is no comprehensive study dedicated to the passive wake-up radios for IoT applications in the literature; from the classification related to the wake-up radio techniques based on previous works, the cross-layer design considerations for potential IoT applications, the opportunities, design challenges, and possible solutions. This survey highlights the major advances in the passive wake-up radio hardware and MAC protocols design. We establish a framework for the design and development of the passive wake-up radio over a cross-layer approach. Our paper also seeks to analyze the opportunities and challenges of the technique, as well as identifying the potential application areas of the passive wake-up radios for the Internet of Things utilization.

### 1.3. Contributions

This paper presents the following.

1. State-of-the-art in passive RF wake-up radio technology, architecture, extensive survey, and classification of the research efforts and recent breakthroughs made in the field.
2. The structure of the passive RF harvesting circuit, offering a critical review of previous works in the RF energy transfer and harvesting techniques.
3. Description of the communication protocols as well as an extensive survey of the existing MAC protocols used for the passive wake-up radios.
4. Outlining the potential Internet of Things applications, research opportunities, and challenges in the passive wake-up radio technology.

### 1.4. Organization of the Paper

The rest of the paper is structured as follows. In Section 2, the circuit design and system architecture of the passive wake-up radios are demonstrated. State-of-the-art in the RF energy transfer and harvesting techniques used for the system is presented and a survey of the existing passive wake-up radio hardware architecture available in the literature is provided in the section. Section 3 discusses the communication principles and reviews the existing MAC protocols used for the passive wake-up radio. In Section 4, the key evolving application areas utilizing the passive wake-up radios are discussed. Section 5 describes the open research issues and practical challenges in the passive wake-up radio and describes the future research direction that can be taken to solve these challenges. Finally, a conclusion for the survey is presented in Section 6.

## 2. Passive Wake-Up Radio

The growth in IoT draws massive interest in the use of RF energy harvesting in making self-sustainable devices realistic [6]. Electromagnetic energy from the RF source is utilized to feed an electronic device remotely [25]. RF harvesting wake-up radios utilize RF energy as their power source. During RF energy harvesting, electromagnetic waves are converted into electricity using a rectifying antenna, or rectenna. The energy is transmitted in the form of electromagnetic radiation through a radio signals medium with a frequency range from 3 kHz to as high as 300 GHz.

In this section, we present the configuration in the passive wake-up radio hardware architecture and the RF energy transfer and harvesting techniques. We first explained the passive wake-up radio design and the circuit used for the RF-based energy harvesting in wake-up radios. We then describe the state-of-the-art in the RF energy transfer and harvesting techniques and finally reviewed the existing passive wake-up radio architecture available in the literature.

### 2.1. State-of-the-Art in Passive Radios

Besides RF energy transfer and harvesting, electric energy is harvested by resonance coupling [47] and magnetic inductive coupling [48]. However, the inductive and magnetic resonance coupling wireless transmission techniques are considered as a near-field [49], whereby the magnetic coupling can be so weak at long distance that the receiver coil’s effect on the sender coil is almost negligible. Although these techniques have high conversion efficiency and power density, the efficiency of power transmission is dependent on the coupling coefficient, which decays as the inverse cube of the distance between the resonators [50]. Moreover, both resonance coupling and inductive coupling need the coils at the transmitter and receiver to be calibrated and aligned. This makes them unsuitable for remote charging as in the case of wake-up radios. However, such constraints are not present in the RF energy transfer. RF energy transfer is considered as a far-field energy transfer technique.

Within the previous decade, low-power transfer using RF has been attracting research attention. These low-power RF energy harvesting circuits are used to power the radios of wireless sensor nodes as well as the wake-up radios. Due to the easy accessibility of the RF energy harvesting source and reduced dependency on environmental changes, its energy supply is more predictable than the other techniques. The RF transmission signal strength is inversely proportional to the distance between transmitter and receiver. Although large amount of ambient RF power is transmitted, the energy at the receiver is usually very low because as the signal spreads further from the energy source, it is attenuated gradually. Therefore, wake-up radios with energy harvesters should be sited near the RF source to scavenge RF energy.

### 2.2. Passive Wake-Up Radio Hardware Architecture

Although passive wake-up radios need power to operate, power is not required to be continuously supplied all the time. This can be achieved by harvesting energy from the incoming RF (wake-up) signal. Thus, more hardware needs to be attached at the wake-up radio to serve this function. This increases the size and complexity of the circuit.

The energy harvesting process, however, causes a delay in the wake-up of the main radio due to the time needed to accumulate enough energy. This increases the latency and reduces throughput in the node, hence affecting the overall network performance. Passive wake-up radios also have shorter ranges than the active wake-up radios, typically by a few meters. The shorter ranges occur due to reduced sensitivity as compared to the active wake-up radio with active receiver components, thus lower the receiver sensitivity. Moreover, the energy harvesting process places extra work on the transceiver in the case where the wake-up signal is sent by the main radio. The transmitter needs to modulate the wake-up signal and transmit for long enough periods (normally for seconds) so that the signal can be detected at the receiving point. More power is thus consumed when the transmitter is active for a longer period.

**Figure 1:**
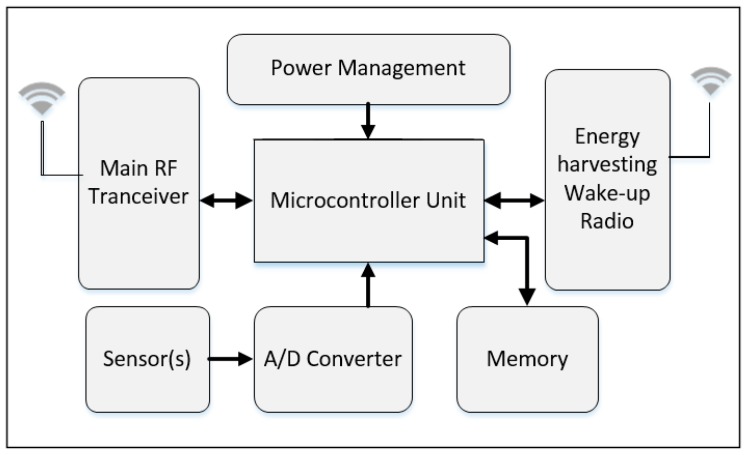
Sensor node with passive wake-up radio.

A generic sensor node with passive wake-up radio is shown in Figure 1. It consists of eight major units: antenna unit, energy harvesting wake-up radio, microcontroller unit, power management unit, memory unit, analog-to-digital converter unit, main RF transceiver unit, and the sensor node.

The wake-up signal is first received by the antenna of the passive wake-up radio and then passed through the energy harvesting process. The harvested power is used to activate the microcontroller unit, which then sends an interrupt signal to wake-up the main radio to exchange data messages.

**Figure 2:**
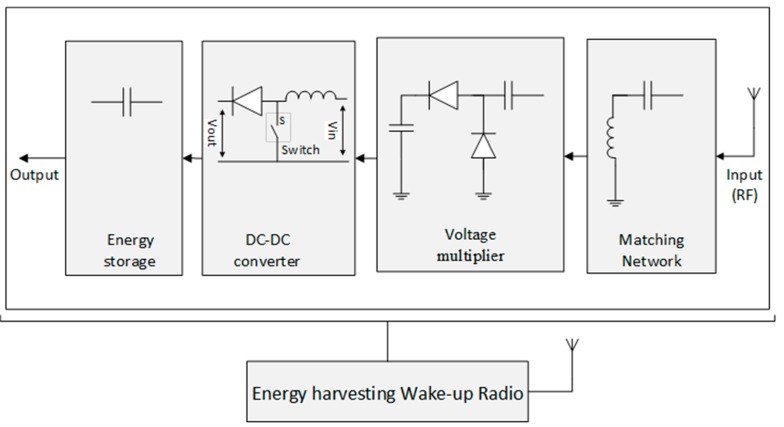
Breakdown of the components for the energy harvesting circuit.

A typical RF energy harvesting circuit block diagram is shown in Figure 2. It consists of four units: matching network, voltage multiplier, energy storage, and the antenna as the RF input. The functions and characteristics of each unit will be described in the next subsections.

The harvested wake-up signal is of different polarization and frequency. The signal is passed through the matching circuit so that the input impedance obtained from the antenna is matched with the wake-up radio circuit load to allow the maximum power transfer to the capacitor. The RF energy is converted to DC voltage in the voltage multiplier and the output DC voltage is passed into a capacitor and stored there. Thus, the performance of the energy harvesting circuit is assessed based on its conversion rate and the available ambient RF energy [51]. However, the RF to DC conversion efficiency and the output DC voltage can be reduced by multiple dissipation and reflection in the terrain, which causes a decline of the available ambient RF energy. Recently, RF energy harvesting circuits are designed to work on multiple frequency bands as opposed to the previous models, which only operate on a single frequency band. These multiple frequency band circuits offer higher dc output voltage because they can harvest the energy from the various RF sources available.

Furthermore, a higher dc output voltage can be obtained by using wide-band harvesters, which can collect several RF signals at a time. However, on the wide frequency band range, it is difficult to maintain the high conversion efficiency and impedance matching. This is due to the mismatch of the diode input impedance and the received RF energy, resulting from nonlinearity of the devices [52,53]. The basic components of the energy harvesting circuits are described below.

#### 2.2.1. Antenna

The passive wake-up radio’s antenna collects energy from the various frequency band of ambient RF source [54,55,56]. The received RF signal is converted into an electrical signal by electromagnetic induction and passed to the matching network for further processing. The antenna can be broadband [57], single-band [58,59], dual-band [60], triple-band [61], and so on depending on the frequency bands in which the antenna operates. For the RF energy harvesting, high radiation efficiency antennas operating at desired frequencies and polarization states, with omnidirectional/hemispherical radiation patterns are preferred, due to the considerably low-power densities.

#### 2.2.2. Matching Network

For maximum power delivery to the rectifier stage and reduced transmission loss from the antenna, it is essential that the antenna impedance is matched with the impedance at the input of the rectifier diode. This process is called impedance matching and it is achieved by using the impedance matching network [62,63]. The matching network consists of inductors and capacitors connected in series or parallel. These passive components are used because they do not add noise to the circuit and do not waste power as opposed to the active components.

The matching network is connected between the antenna and the rectifier block of the energy harvesting circuit. The energy harvesting circuit shows nonlinearity due to the presence of nonlinear components such as the diode. This results in the rectifier circuit impedance to differ from the antenna load impedance and the input RF signal frequency [64], and hence reduces the circuit efficiency. This setback can be addressed by the matching network using components such as inductors, resistors, capacitors, or microstrip lines in ‘*L*’, ‘π’, or ‘*T*’ networks according to the shape in which they are arranged. For example, the values of the components in an L-matched network with a shunt capacitor and series inductor can be obtained using the following equations.

(1) $$B_{C}=\pm \frac{\sqrt{\left(Z_{0}-R_{L}\right)/R_{L}}}{Z_{0}}$$

(2) $$B_{L}=\pm \sqrt{R_{L}\left(Z_{0}-R_{L}\right)}-X_{L}$$

where *Z_L_* is the load impedance. *Z*_0_ is the antenna impedance (typically 50 Ω). *R_L_* is the real part of the load impedance. *X_L_* is the imaginary part of the load impedance.

To match the impedance of the antenna with the diode, the values for the shunt capacitor (BC) and the series inductor (BL) are determined from Equations (1) and (2), respectively.

#### 2.2.3. Voltage Multiplier

To charge the capacitor of the passive wake-up radio, the RF signal from the matching network need to be converted to DC by a rectifier circuit. A voltage multiplier is a specially designed rectifier circuit that changes the alternating input RF signal to a higher output DC-voltage, which is theoretically an integer multiple of the peak AC input. In a two times multiplier (voltage-doubler) for example, a 100 V peak voltage input can produce 200 V DC and 400 V DC for a quadrupler.

The voltage multiplier is a network of capacitors and diodes, which only permit the flow of electron in one direction, placed between the matching network and the energy storage. Based on the Friis transmission equation [65], the antenna gain, RF source power, transmission frequency, and the distance between the transmitter and receiver determines the signal strength, which may result in low conversion efficiency and as such low output DC voltage.

The voltage multiplier circuit helps to boost up the output DC voltage [59]. The most commonly used diode for rectification in passive wake-up radio is the Schottky diode [66] due to its high conversion efficiency at low input power attained from its low built-in voltage. The rectifier can be classified as half-wave or full-wave rectifier based on their conducting characteristics and as single ended topology [67] and differential topology [68,69,70,71] based on their symmetry properties.

#### 2.2.4. DC to DC Converter

To transform the direct current (DC) to a different voltage level, a DC/DC converter is connected at the output of the voltage multiplier. The DC voltage source can be either increased or decreased by the converter depending on the voltage level and converter type. In the energy harvesting circuit shown in Figure 2, the DC/DC converter block is a boost DC converter (also known as step-up chopper). This converter steps up the output voltage delivered to the capacitor. The value of the inductor (L) depends on the output voltage and current requirements.

#### 2.2.5. Energy Storage

A capacitor is connected at the output of the voltage multiplier to store the output DC voltage. The stored power is used as a reserve source when the external energy source is inaccessible or inadequate. This will ensure continuous power delivery to wake-up the main radio whenever it is needed, hence offer smoother and continuous network operation.

### 2.3. Review of the Existing Passive Wake-Up Radio Hardware Design

**Figure 3:**
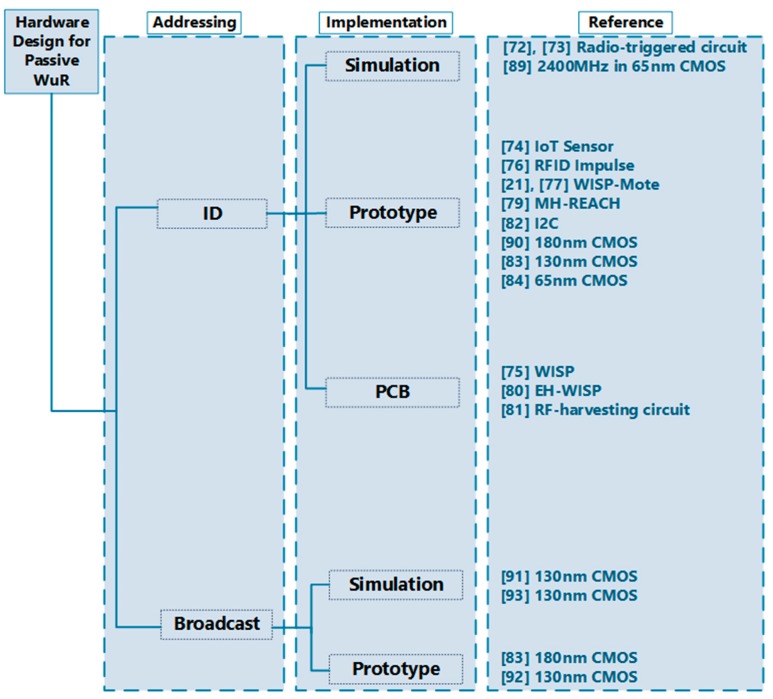
Classification of the previous works on passive wake-up radio hardware design.

In this section, we discuss the prototypes that power the wake-up circuitry using the harvested energy from the RF signal. Figure 3 classifies the existing designs. We categorize and present the literature based on the passive wake-up radio addressing type: ID-based and the broadcast-based. These architectures are achieved using RFID (Radio Frequency ID) or CMOS (complementary metal oxide semiconductor) technologies either by simulation, prototype, or printed circuit boards (PCB). The classification of the existing passive wake-up radio designs is shown in Figure 3. In some passive wake-up radios, the wake-up signals contain a bit sequence for selective node addressing. This is referred to as the ID-based addressing. Although the ID-based increased the size of the signal packet, it reduces false wake-up and thus affecting the overall system energy consumption. In the other addressing types, which are broadcast-based, the wake-up signals are received by the entire neighboring nodes. Broadcast-based wake-up has less data latency as compared to ID-based system, since the receiving node does not need to decode the wake-up packet to check the recipient ID. We summarize the prototypes and their corresponding features in Table 1.

#### 2.3.1. ID-Based Wake-Up Radio Design

The first ever passive wake-up receiver concept [72,73] is presented in 2004 by Gu L. and Stankovic J., which is facilitated by a radio triggered circuit. The circuit collects energy from electromagnetic waves in the incoming radio signal. The receiver then identifies the wake-up signal and distinguishes them from other signals. The design operates at 433 MHz frequency and has the addressing capability. The ID system is designed by applying a multiple-frequency technique called radio triggered ID (RTID), determined by the combination of several frequencies. These combinations are assigned to nodes in the network to serve as group IDs or locally unique IDs of network nodes. SPICE circuit simulation is used to evaluate the technique using experimental data. The design achieved a maximum wake-up range of 3 m and the idle mode power consumption of 145 µW. An optimized, discrete-based wake-up radio for the Internet of Things networks application is designed in [74] with embedded addressing capability. The wake-up circuit is composed of an antenna, an impedance matching network, and a rectifier with a comparator to convert the RF power to a dc voltage. The solution offers a receiver sensitivity of −28 dBm with a power consumption of 39 µW and a data rate of 1 kbps.

WISP [75] is the first microcontroller to be incorporated as part of a passive UHF RFID tag. It is a battery-free system that serves as an enhanced RFID tag. The connected MSP430 programmable microcontroller performs the task of measuring the attached sensors and also the implementation of the EPC Class 1 Generation 1 communication between the WISP and an RFID reader. The model is developed for research purpose by Intel. WISP can achieve an operating distance of 4.5 m.

Another passive system solution is the RFID impulse [76]. RFID tag and reader are attached on the motes. The reader transmits a wake-up signal and the tag collects the RF signal and uses it to transmit an interrupt to the main radio to wake it up. The results show that RFID Impulse outperforms the previous method [75] in terms of energy efficiency and transmission rate. However, their energy consumption analysis does not include the energy consumed by the nodes to wake-up. In addition, the implementation described in the RFID Impulse uses a coil instead of an RFID tag for proof of concept.

In [21,77], the authors integrated the passive tag WISP with the Tmote sky mote to develop a passive wake-up mote, called WISP-Mote. The design used an off the shelf UHF Gen2 Speedway RFID-reader by Impinj [78], which produced the interrupt signal to the Tmote-sky mote. This assembly results in an extended wake-up range of up to 5 m, which is consistent with range on the Tmote sky datasheet. The design supports both broadcast and ID-based wake-ups. The WISP’s microcontroller unit has an output signal of 1.8 V, which is more than the 0.92 V required for an external interrupt to be triggered. After the node is active, the sensor data is transmitted by a 2.4 GHz CC2420 main radio.

In 2015, the same authors also proposed a new passive wake-up system called MH-REACH-Mote (Multi-hop Range EnhAnCing energy Harvester-Mote) [79]. It uses RFID with energy harvesting as well as multi-hop capabilities. To achieve multi-hop, the system has the UHF RFID serving as the WuTx and also a WISP-Mote used as the WuRx which supports ID based wake-up. The system test results shown that for a wake-up signal transmission of 10 s, it consumes the energy of 23.52 J with 9.4 m wake-up range.

In [80], the WISP-mote is integrated with an energy harvesting circuit [81] to further improve the wake-up range. The RF energy harvesting circuit, known as enhanced WISP (EH-WISP) mote, enhanced the wake-up range of the WISP mote. Furthermore, the design of a new wake-up radio node known as REACHMote (Range EnhAnCing Energy Harvester-Mote) is described in [80]. This design can reach a wake-up range of up to 37 ft (11.3 m). It removes the extra hardware necessary for ID-based communication in the WISP mote, making it suitable for broadcast communication only. The dual stage energy harvesting circuit in [81] is composed of a seven-stage and ten-stage design for a reception in low and high input power, respectively.

New strategies for ultralong wake-up radio were implemented in [82] with a new RF energy harvesting circuit to achieve longer wake-up range. Enhance RFID devices were used to realize this using ultralow-power components and a new generation I^2^C RFID chip. The authors were able to come up with three different strategies. First is the WWU (Write Wake-up) strategy obtained from the WWU mode functionality of the Monza X-2K IC. The WWU is fully passive and ID-based. The sleeping Iris mote detects a transition on the serial clock (SCL) line from high to low, which is used as the wake-up signal targeted to the particular Monza X-2K (ID), which is write-accessed by the reader. The second strategy is E-WWU (Enhanced WWU), which is also ID-based, but works on battery. E-WWU exploits the boosted sensitivity of the battery-assisted Monza X-2K and the ultralow power consumption of the TI FRAM MCU to prolong the wake-up range significantly. The third strategy is MWU (Multicast Wake-up), which are fully passive but broadcast-based. MWU uses the RF energy harvesting circuit to produce a wake-up interrupt for the sleeping Iris mote. The mechanism is considered a broadcast-based because any Iris mote equipped with a harvester and within the coverage of the reader is awakened. The longest wake-up range achieved was 22 m with a delay of 198 ms under 30 dBm transmission power.

In [83], the authors described a simple oscillator free wake-up radio design. The proposed system uses the envelope detector and baseband amplifier with a passive RF voltage transformer preceding the receiver. This offers a robust design, which is easy to implement on an ordinary CMOS without the need for external added components. A data rate of 200 kbps and −47 dBm sensitivity was measured with a 2.3 µA current consumption from a 1 V supply.

An energy harvesting power management unit (EH-PMU) was recently presented in [84] to solve the problem of custom wake-up signal needed by wake-up radios, which cannot be produced by a standard-compliant radio [85]. The proposed system initiated the implementation of the backchannel communication from Bluetooth low energy (BLE) [86] compliant to an ultralow power wake-up-radio. The chip fabrication was implemented in a 65 nm CMOS process consisting of a broadband, noncoherent RF front-end [87] and a 32 Hz crystal oscillator. The design harvests energy up to 30 mV and enabled fully passive operation. The systems support RF energy harvesting mode, cold-start mode [88] during start-up and normal boost operation. In [89], ultralow power wake-up receiver (WuRx) based on direct active-RF detection architecture. The receiver is designed through a single-ended topology that reduces the number of circuit components to minimize the system complexity. The 2.4 GHz RF OOK passive wake-up receiver in this design is implemented 65 nm CMOS process. The receiver achieves a total power consumption of 12 µW at −50 dBm sensitivity and data rate of 100 kbps.

#### 2.3.2. Broadcast-Based Wake-Up Radio Design

A 900 MHz passive CMOS-based RF transceiver chip was presented in [90] as WuRx. The microsystem consists of a digital baseband with NVM (nonvolatile memory) and an RF front-end. The RF front-end block comprises a modulator, demodulator, voltage multiplier, bias circuit, a ring oscillator, and a voltage limiter. The experimental result shows that with an oscillation frequency of 2.07 MHz, 2.64 µW of power was consumed with a sensitivity of −17 dBm.

A fully passive 868 MHz wake-up radio with high sensitivity is presented in [91]. The radio front end is designed on a 0.13 µm CMOS including an antenna, voltage multiplier, impedance matching network and data slicer. For a 100 kbps data rate of the wake-up signal, the system is able to achieve a sensitivity of −33 dBm.

Similarly, the authors in [92] presented another passive wake-up radio, which is also implemented in 0.13 µm CMOS process. The design is developed for wearable medical gadgets, operating using FSK modulation at 8 Mbit/s data rate and a frequency of 915 MHz. The results show that sensitivity of −53 dBm and −78 dBm were achieved for the WuRx and the main receiver respectively. The power consumed by the main receiver was 640 µW and the transmitter uses 1.4 mW from the 1.2 V supply. The wake-up receiver dissipates 0.2 µW power at a sensitivity of −53 dBm.

Authors in [93] described a 2.45 GHz passive wake-up circuit, which consists of an antenna, rectifier and matching network. The circuit design was optimized to achieve improved input voltage and maximum sensitivity and conversion efficiency. An output voltage of 1.8 V was achieved for a received RF power of −23 dBm. The rectifier circuit was implemented in UMC 180 nm CMOS process.

Recently, the work in [94] studies the fundamental performance limits of the passive wake-up radios and shows the advantage of using the PWUR when a number of nodes are relatively small. Many other studies have proposed the wake-up radio schemes that are not fully passive. For example, the study in [95] proposed an ultralow power wake-up radio based on direct active RF detection with power consumption of just 4.5 μW at 2.4 GHz, sensitivity of −50 dBm, data rate of 200 kbps, and 4.5 μW consumption from a 0.8 V supply voltage. The work in [96] fabricated a circuit in a 0.18-μm CMOS process with the sensitivity of −80.5 dBm and consumed 6.1 nW. The high sensitivity is realized by using a passive pseudo-balun envelope detector. Another similar design [15] achieved −60 dBm sensitivity while consuming 166 nW of power. To implement the sensitivity, a passive voltage-boosting network comprising of a high-quality factor (high-Q) piezoelectric MEMS resonator is used instead of an inductor to amplify the received voltages applied to a CMOS rectifier. Table 1 shows the performance characteristics and properties of the different passive wake-up radio design.

### 2.4. Discussion of the Passive Wake-Up Radio Parameters

A range of hardware design parameters affects the overall performance as well as the power consumption of the passive wake-up radio. In this section, we discussed how these various parameters affect the different reviewed prototypes in Table 1.

#### 2.4.1. Hardware Options

The RFID-based is preferred compared to the other technologies in the application of passive wake-up radios because of its easy implementation. RFID-based designs usually consist of over-the-counter devices. The CMOS is also utilized in the design because of its low power consumption. Although the wake-up ranges are not given in almost all the CMOS designs, we can infer that they have higher wake-up ranges than the RFID based schemes from their higher sensitivity values. This is because the communication range is always directly proportional to the receiver sensitivity. The design in [82] has the highest communication range, achieving 11 m in the fully passive design and up to 22 m when it uses battery assistance to boost sensitivity. The RFID based circuit presented in [80] can be said to be an outstanding design having the least input circuitry power-up voltage with relatively high wake-up range of 11 m and a strong output DC voltage of 2.074 V. Conversely, the power dissipation values are not given in all the RFID designs due to the obscure nature of the commercial RFIDs, which does not allow easy measurement of the parameters. This is why the CMOS-based option is preferred for research-based designs.

#### 2.4.2. Input and Output Voltage Requirements

The *V_in_* column describes the minimum voltage required to power up the wake-up radio circuit. It is a critical design factor that needs to be considered for varying RF environments. Low turn-on voltages can be designed for weak RF environments while only high *V_in_* voltage values can work well in strong RF environments. The *V_out_* is the output DC voltage obtained from the harvester circuit. This voltage charges the energy saver so that sufficient power is available to trigger the interrupt, hence activate the main radio. The ratio of the minimum *V_in_* to V*_out_* is generally lower in RFID designs than the CMOS, meaning that lower input voltage can produce a higher output voltage in the RFID designs than in the CMOS designs. Lower wake-up time is preferred, as the designs with high wake-up time, such as [21], require 726 ms, which increases latency in the network.

#### 2.4.3. Sensitivity

Receiver sensitivity is a measure of how well the receiver performs and is defined as the power of the weakest signal the receiver can detect in −dBm. Higher sensitivities result in longer wake-up range. However, higher receiver sensitivity also translates to increased power consumption. Since power consumption, sensitivity and wake-up range are important features for effective passive wake-up radio performance, the trade-off between these factors need to be considered during the circuit design. However, from Table 1, the values in the dissipated power column are not changing relative to the sensitivity due to several design factors. The design in [90], for example has a low sensitivity of −17 dBm but a relatively high power dissipation of 2.64 µW, as a result of other parameters, such as the modulation scheme. However, in [84] the wake-up radio has relative high sensitivities of −56.5 dBm and −39 dBm, and the power consumption is measured as only 236 nW and 104 nW, respectively. Although the power dissipation is higher when the sensitivity value is higher, the very low power consumption can be explained by the efficiency of the advanced design techniques utilized in the protocol, such as the back-channel communication and Bluetooth low energy (BLE). The BLE offers the lowest average radio power that can communicate directly to a mobile device [86]. Back-channel modulation controls the BLE standard compliant radio to produce signals that a passive wake-up receiver can demodulate to recover the back-channel information.

#### 2.4.4. Data Rate and Modulation Scheme

The values for the maximum data transmission rate were selected based on a bit error rate (BER) at the receiver. Shannon capacity draws a theoretical bound for the data rate as a function of the signal-to-noise SNR ratio at the receiver side [97]. The very high data rate at small transmit power produces many errors. Therefore, higher data rates require higher transmission power, which consequently results in higher energy consumption. Therefore, the data rate does not have a direct effect but can be a function of the transmission power, in relation to power consumption. For instance, the design in [84] has a low data rate and also very low power consumption. The design in [83], however, has a relatively high data rate of 200 kbps with a relatively low power consumption of ~2.3 µW.

The frequency-shift keying (FSK) and amplitude-shift keying (ASK) modulation schemes are utilized in different wake-up radio designs. However, the on–off keying (OOK) modulation technique is preferred and more often used for the passive wake-up receiver. In the passive wake-up radio designs, the power consumption value of 2.3 µW measured in [83] is the highest recorded value in all the studies reviewed in our work utilizing the OOK scheme. The OOK is an envelope detection-based technique with low power dissipation feature. This makes it suitably embraced for the passive wake-up radios.

#### 2.4.5. Operating Frequency

The operating frequency used is also a factor that affects power dissipation. The power consumption is increased when a higher frequency is used, since the power consumption *P* is directly proportional to the operating frequency, given as *P = CV*^2^*·f*, where *f* is the operating frequency, *C* is the load capacitance, and *V* is the voltage. Therefore, more current will be required in radio circuits operating at a higher frequency to attain equivalent performance as those running at a lower frequency. However, due to other factors the frequency alone does not determine energy consumption. In our table, the highest frequency used is 2.4 GHz and the lowest is 0.433 GHz. The 2.4 GHz, 0.915 and 0.9 GHz frequencies are the most popular in Table 1. However, the lower (sub-GHz) frequencies are now being preferred for the wake-up radios by most designers than the higher frequencies. This is because, the attenuation rates increase at a higher frequency. That is to say the sub-GHz signals last longer than the 2.4 GHz signal before it weakens. Based on the Friis equation [65], at 900 MHz the path loss is 8.5 dB lower than at 2.4 GHz, which means the 900 MHz operating transceiver has a 2.67 times longer range than that operating at 2.4 GHz.

## 3. Wake-Up Radio Communication Protocols

The passive wake-up radios help in mitigating the energy consumption of the sensor nodes at the hardware level as we explained in the previous section. However, this hardware needs MAC protocols to control its communication functions. These strategies are employed to coordinate the wake-up radio activities as well as its trigger functionality while simultaneously putting the main radio in a deep sleep until when it is required to be active. The MAC layer mainly coordinates how broadcast channels are shared between nodes to prevent collisions as well as reducing delays in accessing the channel, increasing energy consumption efficiency and guaranteeing fairness between the nodes. Disadvantages of RF energy harvesting include the decreased wake-up range as well as increased latency. This occurs due to the time required to harvest sufficient power from the RF signal.

A sensor node with passive wake-up radio utilizes two channels; the channel for the main radio and that of the wake-up radio communications. The wake-up radio channel can be classified for use in the two basic wake-up communication types: the single hop and multi-hop wake-up networks. In this section, the basic design for RF energy harvesting network and communication protocols is described. Subsequently, we reviewed the existing MAC protocols and compared them.

### 3.1. Single Hop and Multi-Hop Wake-Up Network

In a typical sensor network scenario, the wake-up signal is sent by either the wake-up radio or main radio to a neighbor passive node. The wake-up receiver of the receiving node will accumulate and harvest enough energy from the signal to become active and then trigger an interrupt to wake-up its main radio. The main radio is turned on to perform the required communication function and the wake-up radio will immediately return to sleep for a single hop communication network. On the contrary, the wake-up radio in a multi-hop network will have to retransmit the wake-up signal to a neighboring node before going back to sleep. Hence, the wake-up radio in the multi-hop network needs to have specialized hardware functionality.

#### 3.1.1. Wake-Up Transmission

**Figure 4:**
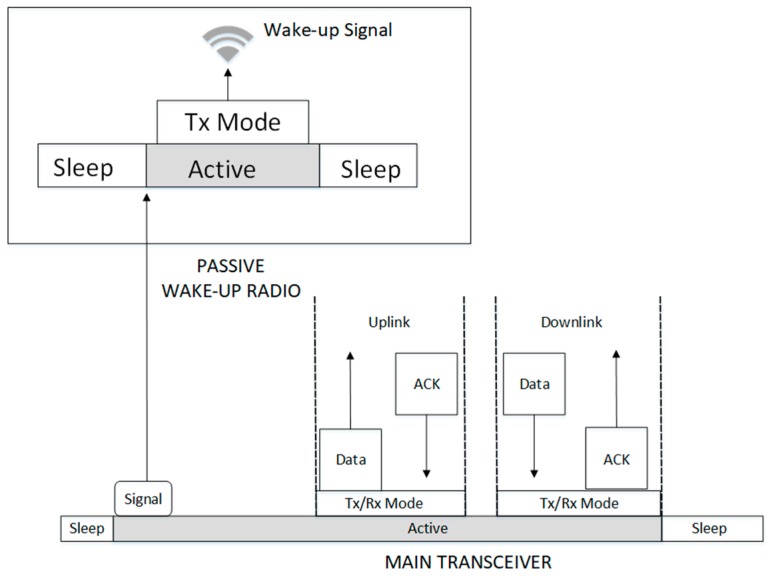
Transmission process.

This is the wake-up radio, which is transmitting the wake-up signal at the given instance. This can be used to initiate communication or send a wake-up ACK/NACK. The wake-up signal transmission for communication initiation can be started by the message sender or receiver depending on the intentions of the initiator; either to send or request for data, respectively. The process is started by the main radio, which becomes active according to schedule and sends an interrupt signal to its wake-up radio to activate it. The wake-up radio generates the wake-up signal and sends it to the neighboring nodes, while the main radio remains active and ready to transmit or accept data as shown in Figure 4. An example of the wake-up transmitter is the RFID reader in the RFID-based passive wake-up radio.

#### 3.1.2. Wake-Up Reception

**Figure 5:**
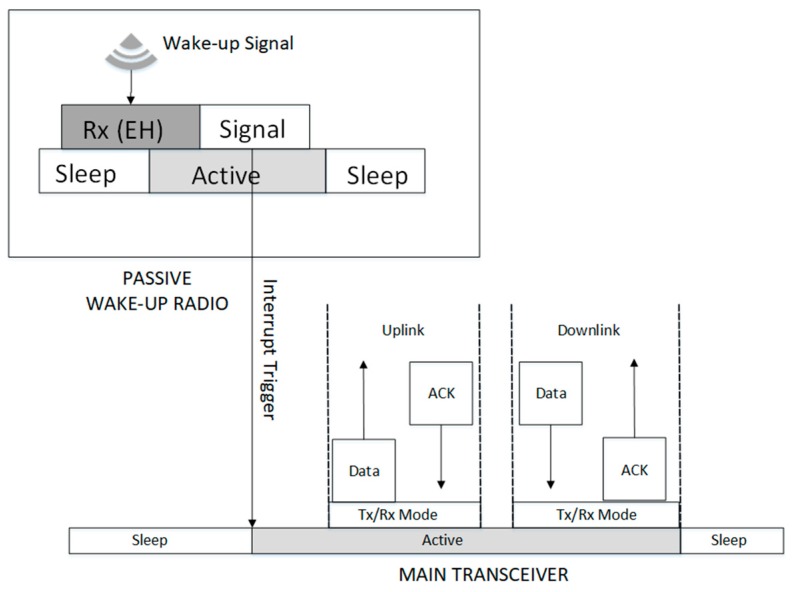
Passive wake-up reception process.

The energy harvesting process takes place at the wake-up receiver. While the entire node is in deep sleep, the wake-up receiver taps and harvests enough energy from the RF signal of the incoming wake-up message. Depending on the signal type, the wake-up radio checks the signal packet to determine if the wake-up call is destined to it. Once it is confirmed that the packet is meant for the radio, it uses the energy from the RF signal to become active and transmit an interrupt signal to wake-up the main radio. After activation, the main radio sends data and waits for an ACK and/or receives the data and reply with an ACK if the reception was successful and then returns to sleep. The wake-up radio will return to sleep after sending the interrupt trigger signal. The passive wake-up reception process is shown in Figure 5.

#### 3.1.3. Wake-Up Multi-Hop (Relay)

**Figure 6:**
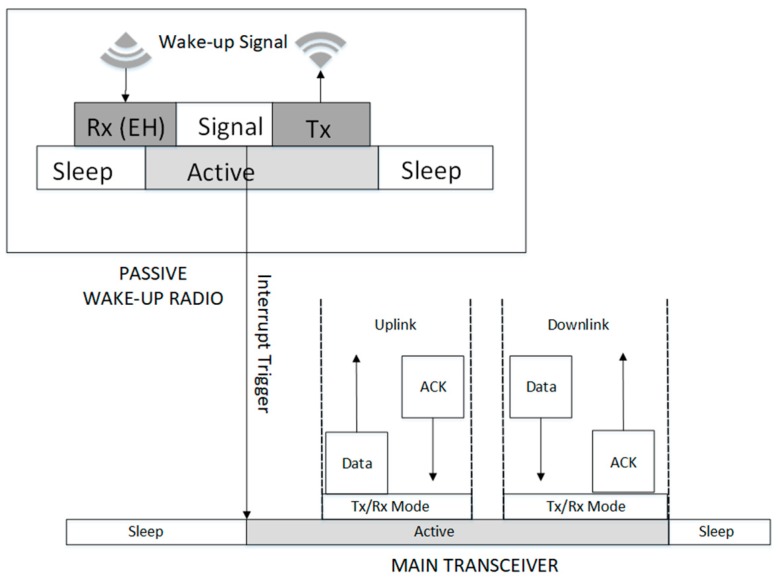
Wake-up relay process.

This type of wake-up radio is used in multi-hop networks. It can simultaneously serve as a wake-up signal receiver and transmitter, and hence can relay the wake-up signal to a next hop. The wake-up radio check to determine the wake-up packet is intended for it and taps the wake-up signal to harvest enough energy to become activated. The wake-up radio sends the interrupt trigger to the main radio to perform its sending and/or receiving function. However, the wake-up radio relays the wake-up call to the next hop address when it reads that the address on the signal packet is destined for another node. The relay of a wake-up signal is performed by the wake-up radio transmitter without waking up the main radio. If the node (known as an intermediate relay node) detects an event where a signal is to be forwarded, it packs the signal information into a packet, and delivers the packet to a next hop via multi-hop relaying. After forwarding the wake-up signal, the node usually sets an internal timer to avoid starting a new data transmission before the packet forwarding from the source to the destination node is expected to complete. The wake-up multi-hop process is shown in Figure 6. The nodes need to have hardware with transceiver capability that can receive and send a wake-up signal.

### 3.2. Passive Wake-Up Signal Packet

**Figure 7:**
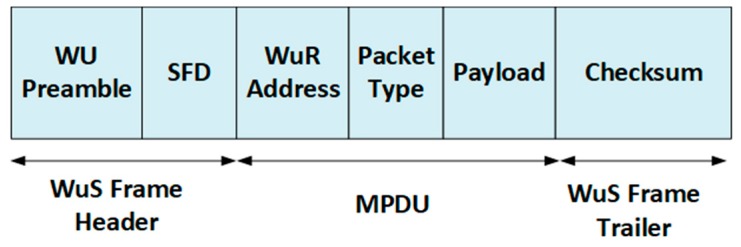
WuS frame structure.

Here, we describe a typical passive wake-up signal data frame and its composition. Although various technologies have different packet structure, we attempt to illustrate a generic structure which meets the standards and compliance requirements of diverse applications. Efforts [98] have been made in recent times to develop a standard packet structure for medical applications. The packet of a generic wake-up signal is shown in Figure 7.

#### 3.2.1. Wake-Up Signal Frame Header

The wake-up preamble is the first part of the frame header, which is responsible for generating the wake-up signal. The bits in the preamble are responsible for the transmitter and receiver bit interval synchronization. The SFD (start frame delimiter) allows the wake-up radio to know the actual frame header position and when exactly to start frame decoding.

#### 3.2.2. MPDU (MAC Protocol Data Unit)

This is the main body of the wake-up signal data link layer frame, which consists of fields for address, packet type, and payload. The destination wake-up radio address is contained in the address field. The field for packet type identifies whether the packet being sent is a beacon, information, ACK/NACK, command, or stream. The payload field contains the actual sampled data.

#### 3.2.3. Wake-Up Signal Frame Trailer

The frame-check-sequence (FCS) is performed on the WuS frame to check the integrity of the data using cyclic redundancy check (CRC) or FEC.

### 3.3. Review of the Existing MAC Protocols

Several strategies are employed to coordinate how broadcast channels are shared between the passive wake-up radio nodes in a network. These strategies aim to prevent collisions with minimal delay in accessing the channel as well as higher energy efficiency and fairness between the nodes.

**Figure 8:**
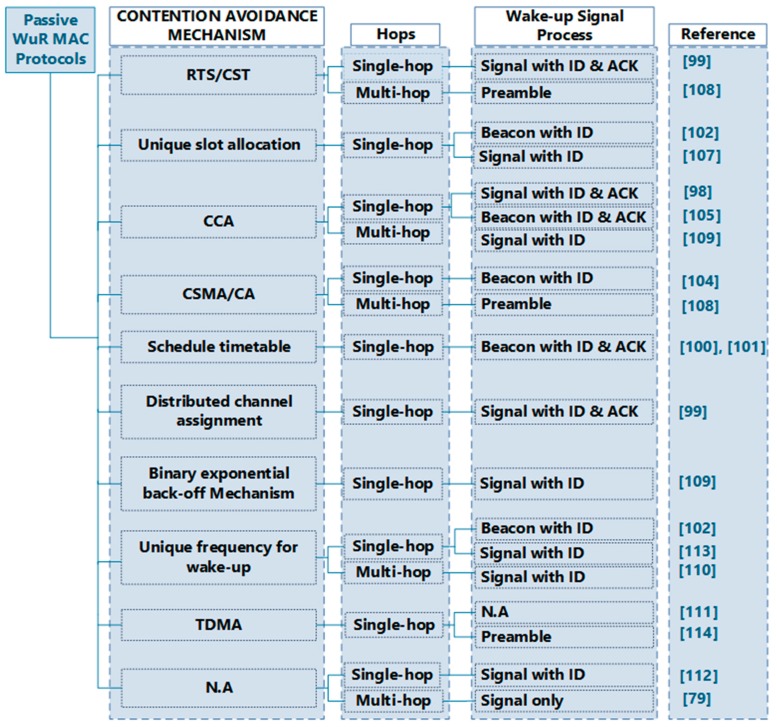
Classification of the previous works on passive wake-up radio MAC protocols.

In this section, we identify all the strategies that have been applied in the passive wake-up radio networks. These collision avoidance strategies are categorized in Figure 8. We also recognize the MAC layer protocols available in the literature and categorize them based on the strategies employed. Table 2 gives a summary of the existing protocols.

Dual radio architecture for multi-hop networks was proposed in [99] to minimize the effects of ultrawide band (UWB) acquisition time. An UWB-based radio with a physical layer that is based on a single band impulse radio was used as the main radio. The secondary radio is an RF-based passive wake-up radio, which provides control signaling using an out of band narrowband control channel. To reduce the signaling complexity and avoid a collision, the system uses the dynamic channel allocation concept. The simulation results have shown that the technique achieved improved network throughput and reduced latency.

In [100,101], another MAC protocol for passive wake-up radio [73] is presented. The protocol is designed for single hop star network for wireless body area networks (WBAN), which handles the normal and emergency traffic types. When there is an emergency, the BAN Nodes (BN) becomes active and transmits a wake-up message to the BAN network controller (BNC). The BNC replies to the BN by sending an ACK and then a beacon for resource allocation purpose. The BN then transmits its data to the BNC and the session is finished when the BN received an ACK from the BNC. For the normal traffic, the BNC wakes up according to a TDMA based schedule and initiate the communication. It sends the wake-up signal to wake-up the BN. The BN replies by a negative ACK (NACK) if it has no data packets to send, otherwise it will send an ACK to the BNC. The latter then replies by a beacon which contains the channel, synchronization, priority, and slot information. The data is then exchanged and the communication ends with an ACK from the BNC. The method is able to achieve increased energy efficiency and reduced latency.

Another protocol proposed in [102] called very low power MAC (VLPM) integrates the passive wake-up transmitter [81] and receiver [103]. The scheme is designed for single-hop WBAN with the wake-up transceivers mounted on both the BNC and BN nodes. The BNC sends Res-WUP (i.e., the wake-up packet together with the resource allocation and other information). The Res-WUP is received by the BN and activates the MCU to interpret the information to know whether it contains the nodes’ address. The MCU goes back to sleep after the interpretation, and the main radio is only woken up if it is the intended receiver. The BN replies by sending the data, and then the BNC again replies by IMM-ACK. The BN only initiates a wake-up packet to the BNC in case of emergency. The BNC then replies a RES-ACK, and the BN subsequently begins to send its data packets. After this, the BNC sends an IMM-ACK.

Passive synchronization-based energy-efficient MAC (PSMAC) [104] is another protocol, which works by broadcasting a synchronization frame to activate the radio triggered circuit. The synchronization frame is embedded within the network information from which the nodes scans to discover their neighbors. PSMAC supports the various M2M QoS requirements as well as mobility while efficiently utilizing the energy. The protocol also offers preemptive and fast slot allocation by placing the contention access period before the beacon period. It supports diverse data types of the M2M by making its superframe to have both contention-free and the contention-based schemes along with a priority. A separate channel called SC is dedicated for synchronization, which uses a CC2420 radio hardware switching capability. The network is initialized by the coordinator performing a passive scan of the SC for an SF. If the SF is received, the coordinator schedules its superframe after the SP duration from the time instance of the received SF. Otherwise, it starts its superframe immediately. Results have shown that the PSMAC protocol is fast, energy efficient, supporting the diverse QoS requirements of M2M with low latency.

WUR based Transmitter Initiated Cycled Receiver WUR-TICER [105] combines the wake-up radio based on the TICER [36] protocol with the energy harvesting concept. Communication is initiated by a wake-up beacon (WUB) from the transmitter to the passive wake-up radio of the receiver. The WUB and data packets are both transmitted on the same channel using the main radio of the transmitter, which results in a low rate of packet reception. The protocol achieves a significant reduction in energy consumption and up to 82% improvement in throughput.

A smart power unit coupled with a nanowatt wake-up radio receiver is developed in [106]. The hardware unit can be used for harvesting various energy sources, such as RF, wind, solar, etc. The ultralow power microcontroller in the system performs MPPT (maximum power point tracking) and optimized charging of the super capacitor at the highest efficiency. The power unit uses the serial interface (SPI) to communicate with the supplied node.

DoRa [107] is a cross-layer protocol for passive wake-up radio architecture in single hop star networks. In this scheme, communication is always initiated from the base station by periodically sending a wake-up call to each node including its Mac address. The node replies by sending a data message to the BS. The BS activates transmission mode when it needs to send a wake-up call and reception mode is activated after immediately after sending the wake-up call. The scheme helps in eliminating the possibility of collision in the system. To confirm the wake-up call, the receiving node must authenticate the MAC address in the wake-up call. The signals received power must also be strong enough throughout the wake-up preamble, which is equivalent to a continuous voltage from the wake-up radio.

Other symmetric multi-hop enabled protocols are proposed. A new multi-hop passive wake-up radio MAC protocol known as radio triggered sensor MAC (RTM) protocol is proposed in [108]. In this scheme, both the main and wake-up radio uses the same medium. The transmitter has first to find a free channel using the CSMA/CA protocol and transmits the wake-up signal. After waking up the receiver, the transmitter will then send an RTS and await CTS. It resends the RTS if it does not receive CTS after some certain time and will give up and return to sleep after five trials. The transmitter sends its data after receiving CTS and awaits ACK. It retransmits the data after a certain time if the ACK is not received, and then return to sleep after receiving the ACK. On the receiver side, it first harvests the energy from the received signal to wake-up the node and waits for the RTS failure of which it returns to sleep. It verifies if the RTS is destined to the node, if not it returns to sleep. It then sends a CTS and waits for data, after which it waits for ACK. It then goes to sleep after receiving the ACK; otherwise, it retransmits the data until the ACK is received. Thus, it sends the wake-up message to the nearby nodes and then selecting the right receiver by exchanging RTS/CTS wastes much energy and reduces the energy efficiency. There may also be increased latency due to the utilization of single channel by both the main and wake-up radio, which results in one having to wait for the other to finish using the channel before it can transmit. The protocol avoids idle listening by employing node time-out and also avoids overhearing by making nodes go to sleep after they overhear an RTS or CTS packet that is not meant for them.

Since both RFID impulse scheme [90] and IEEE 802.15.4 operate in the same ISM band, the IEEE 802.15.4 radio can be used as a general-purpose radio and RFID reader. Hence these technologies are designed to operate together as a nonbeacon passive wake-up MAC protocol in [109] for multi-hop networks to reduce idle listening in. For a node that intends to transmit data in this scheme, the sensor output triggers an interrupt to wake-up the microcontroller unit (MCU). The MCU wakes up the main radio and the main radio performs a random byte slot clear channel assessment (CCA). It transmits the data if it can find a free channel; otherwise, it will return to the idle mode and listen further. For the reception, the node is wakening up from deep sleep when its RFID tag is triggered which turns on the MCU. The MCU turns on the radio, which again listens to the channel in the random byte slot. It listens for a maximum of up to ~17 ms in which if there is no packet, the MCU goes back to sleep.

A similar multi-hop supporting MAC protocol for passive RFID based wake-up radio is the MH-REACH protocol [79]. The nodes functions either as a multi-hop node, which sends a wake-up signal to other node or as an edge node that only receives the signal without relaying to any other node. A mobile sink moves around the nodes and initiates the wake-up signal transmission, which wakes up the entire nodes within its range. The nodes that are activated will send their data to the mobile and otherwise, in the case of a multi-hop network, another wake-up signal is further transmitted to the nearby nodes. All nodes return and remain asleep after performing the task with their passive wake-up radios scan for an incoming signal. The waking up of all the nodes within the broadcasted wake-up signal increases the overhearing overhead which reduces the energy efficiency.

SLAM [110] is another passive MAC protocol proposed, which reduce energy consumption due to listening by up to 30 to 129 times, while at the same time providing security in the network. The security is achieved by assigning some nodes as guard nodes, which supervise the network traffic and identify malicious activities by nodes. These guard nodes remain asleep and are activated by the passive wake-up radios when needed.

A method for enhancing WSN performance and reducing its carbon footprint is proposed in [111]. Three schemes—wireless energy harvesting, wake-up radio, and error control coding (ECC)—are incorporated and used to maximize the WSN lifetime. The proposed scheme reduced the energy consumption and the carbon footprint in the nodes. In [112], the authors developed an RF-based out-band wake-up mechanism for aerial to ground data transmission. The RF is used as the wake-up channel. The receiver, AS3933, has three wake-up channels, each connecting to an antenna, and any antenna that received the correct wake-up pattern may trigger the wake-up.

An energy-efficient joint downlink and uplink passive wake-up solution for IoT devices over cellular networks was designed in [113]. In this solution, a deep sleep state similar to power saving mode (PSM) by 3GPP is defined. However, the device under this technique will not leave the PSM upon expiry of the timer, but instead upon receiving an explicit RF wake-up signal from its serving BS. A passive wake-up receiver scheme called wireless-powered wake-up receiver (WPWRx) is also proposed in [114]. In the proposed scheme, battery consumption during the listening period is eliminated by transferring wake-up data and power for the WPWRx using time switching. Each WPWRx functions as either a wake-up signaling receiver or an energy receiver in time switching manner at any given time. The scheme can be utilized for a range of applications such as wireless sensor actuator networks, machine-to-machine communications, and the IoT. Table 2 provides a comparison and summary of the MAC protocols that are utilized for passive wake-up radios.

### 3.4. Analysis of Reviewed Mac Protocols

The key function of the MAC layer is channel access coordination among nodes to prevent a collision. This is achieved by employing various collision avoidance and prevention mechanisms. However, problems arise in the channel allocation when the appropriate technique is not applied. The wake-up call may also be overheard by an unintended node. However, these problems are mostly concessions made by protocol designers to achieve improved values in other essential parameters such as reduced latency, energy consumption, etc. The CSMA/CA (carrier sense multiple access/collision avoidance) scheme has been used more in the passive wake-up radio MAC protocols in Table 2. In CSMA/CA, the carrier is sensed before transmitting. The CCA (clear channel assessment) scheme is also utilized in many other protocols for collision avoidance. The RTS/CTS (request to send/clear to send) handshake mechanism was used in [99] and together with CSMA/CA in [108]. This mechanism prevents collision arising from hidden node problems. TDMA scheme assigns one time slot of the whole cycle unit to each passive wake-up radio to handle the multiple access control in [111]. Each time slot should have a length at least longer than the required time for a successful wake-up packet transmission. Compared to TDMA, using CSMA/CA scheme as in [104] does not need the coordinator to reserve a time slot for a specific passive wake-up radio. CSMA/CA provides a more dynamic and efficient way to make use of the channel, especially when the slot length is much longer than the actual data transmission time. However, when the number of nodes increases, especially when too many nodes start CSMA/CA process simultaneously to compete for the medium, performance of the CSMA/CA reduces as TDMA prevents collisions more perfectly than CSMA.

Different techniques with easier implementation are used in other protocols. For example, the design in [100,101] maintains a table for the wake-up schedule. The network coordinator node maintains the wakeup schedule in a table for every passive wake-up radio node in the network. Use of wake-up table by the coordinator node is easy to implement and saves a significant amount of power as all BNs in the network remain in the sleep state (i.e., switch off the main transceiver) until it is woken up by to transmit or receive data. In [107], a unique slot is set for the wake-up packet, while in [110] a unique frequency is set for the wake-up packet transmission. In [109], the CCA is used with a binary exponential back-off mechanism which schedules retransmission after a collision happens.

The in-band channel, which sends the wake-up signals on the same channel with the main data channel is used in protocols [105,108]. This method reduces the complexity and implementation costs, but has lower resistance to jamming attacks to the communication stack and increases the probability of collision. Therefore, during the channel design for multiple sensor node applications, which requires high reliability and low latency such as in medical applications, the in-band channel should be avoided. The out-of-band, which is more popular and used in seven out of the eleven designs send the wake-up signal on a dedicated channel, separate from the main data channel. If separate channels are utilized, the cost increases; however, resistance to jamming attacks increases and energy consumption due to overhearing decreases. From an energy perspective, the out-of-band approach is more efficient. For instance, the out-of-band channel is more suitable for the proposed concept in [112] that emphasizes opportunistic aerial–ground sensing. However, in [99,110], multiple channels are utilized for communication of the wake-up signal. Multiple channels allow multiple nodes to transmit the wake-up signal through different channels simultaneously, which increases throughput and helps mitigate interference.

The destination node ID is included in the wake-up signal of the protocols in the majority of the reviewed protocols. The ID-based signal is essential in periodic or query based data collection applications such as humidity or temperature level monitoring, where a single sink node aggregates the data of multiple sensor nodes. The data collector can request the data from a particular node by simply sending the wake-up signal with the target node ID without waking up the other nodes. However, the protocol in [79] which is for event-driven applications where events take place infrequently, such as forest fire detection or surveillance system whose duty is intrusion detection, the wake-up call is sent as a signal only without ID. The sensor devices wake-up asynchronously to exchange their data whenever there is an event to report. The protocol in [108,114] encapsulates and send the signal together with the preamble signal. The preamble sequence is used for time synchronization by the wake-up radio. For instance, in [114], the preamble sequence indicates the wake-up signal arrival time. However, repeated preamble needs to be received before determining the exact wake-up signal arrival time, which may result in misdetection and as a result add extra delay. Therefore, the preamble-based signal method will not be suitable for applications that require high reliability and low latency, such as medical and security applications. Some other protocols send the wake-up signal along with the beacon frame. Furthermore, the protocols in [99,100,101,102] require acknowledgment of the wake-up signal, indicating that the main radio of the receiver node is actively listening, before the transmitting node begins to send its messages. This further reduces energy consumption and increase reliability.

The test-bed implementation of wake-up radio MAC protocols used in [79] is more realistic, making it more valid and accurate than the simulation. However, evaluation of the other ten of eleven protocols in Table 2 is implemented by computer simulation. A major setback faced in the passive wake-up radio MAC protocol design in [101,102,110] is the delay arising from the time needed to harvest enough energy to wake-up. This increased the wake-up latency, and hence the overall network delay.

## 4. Key Application Areas for Passive Wake-Up Radio

Smart technologies propelled by wireless sensor networks (WSN) are employed in various applications to monitor and control different conditions ranging from natural, environmental and personal health features. In this section, we discuss the key evolving application areas where the passive wake-up radios can be utilized.

### 4.1. Remote Environmental Monitoring

**Figure 9:**
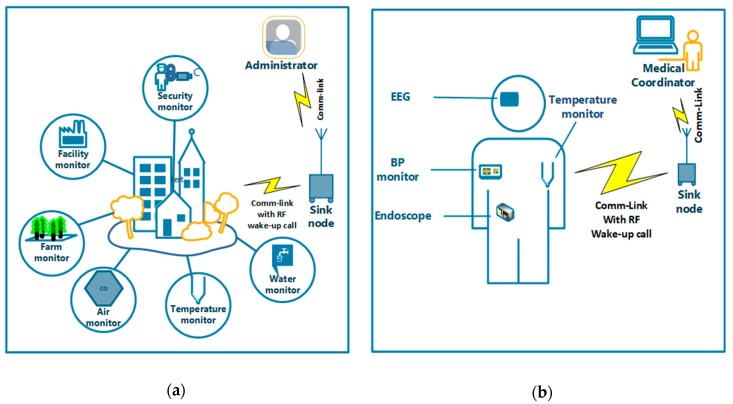
(**a**) Description of passive wake-up radio for remote environmental monitoring. (**b**) Description of passive wake-up radio for medical applications.

Environmental monitoring performed by wireless sensor networks (WSN) is suitable to showcase the capability of a passive WUR technique [5]. It is a combination of a large number of sensor nodes deployed all over a city or rural location to assist humans in easy management and administration of such an environment [115]. However, these sensor nodes need to have a stand-alone energy supply to offer continuous transmissions and ideally without the necessity for a schedule battery change. An example of remote environmental monitoring is shown in Figure 9a.

For instance, the work in [104] proposed three polling service-based MAC scheduling schemes, known as PSMACs, which have radio triggered hardware attached to the node. The scheme supports diverse QoS as well as mobility, which shows that the protocol can be implemented for environmental monitoring. The protocol features demonstrate the feasibility of passive wake-up radio in environmental monitoring, whereby the mobile node can move around the environment to collect data from the sensor nodes. The sensor nodes are equipped with the passive wake-up radios so that they remain in a deep sleep and only wakes up to send data after receiving a wake-up call from the mobile sink. Relative high data rates and communication range as well as robustness and energy efficiency are other features of the passive wake-up radios, which are required in the implementation of the environmental monitoring.

Wildlife monitoring is another potential environmental application of the passive wake-up radio. Animals are equipped with sensor nodes such as using a collar sensor. Data collectors (sink nodes) are placed at strategic locations where the animals are expected to gather or move around. The radio on the animals is awakened the moment they get within the data collector range, and the radio starts transmitting the sensed data. In [77], the authors simulated a similar scenario in a 1000 m × 1000 m area with a data collector at the center. The data collector is set to continuously send out the broadcast-based wake-up call. The monitoring system using this passive wake-up process resulted in a significant reduction in the energy wastage in the sensor nodes, and hence the overall energy efficiency is increased.

The protocol in [112] is designed primarily for wireless aerial–ground sensing network for remote environmental monitoring applications in large-scale geospatial or challenging spaces. Likewise, the ID-based enhanced WISP mote (EH-WISP) mote design in [80] is a suitable candidate for periodic (for event-triggered and nonevent-triggered) environmental monitoring applications. With the enhanced wake-up range of up to 5 m, the MH-REACH can be used for indoor living monitoring and greenhouse monitoring. This protocol utilizes its ID capability to allow for a single sink node to wake-up and aggregate the data of multiple sensor nodes individually, without having to wake-up all sensor nodes within its range.

### 4.2. Medical Applications

Medical application in the form of wireless body area networks (WBAN) is a recent revolutionary sensor network technology used to monitor the human body for both medical and non-medical purposes. High energy efficiency, reliability and variable data rates are thus required in WBAN since emergency and life-critical data is handled. The passive wake-up radio application in the medical field is shown in Figure 9b.

Communication in WBAN is either on-demand for sending normal data, usually initiated by the external sink node or event-driven for sending emergency data, initiated by the sending node. However, long ranges are not necessary for medical applications [116] since the sink node is mostly within the same facility with the sensor nodes, but reducing energy consumption while idle is very important. These make the passive wake-up radios suitable for the medical application. The passive wake-up protocols proposed in [100,101,102] are designed specifically for the WBAN. Other protocols, such as the passive wake-up radio protocol described in [64,108] have the criteria that match the WBAN requirements with ultralow power consumption and addressing capability. A recent design presented in [117] called W2B-IoT, implemented and validated a prototype of a WuR-enabled IoT with a two-tier end device to IoT server connection via BLE and LTE. The solution is targeted at a 5G IoT scenario where battery powered massive IoT devices do not support direct 3GPP connections. This fits well the data transmission range of BLE for indoor applications and can be applied in health-care scenarios.

### 4.3. Smart Grid

Smart grid is a new sensor technology which adds communication, monitoring, control, and analysis capabilities to the electricity grid to improve the electricity supply chain. It is an extension from the power generation station to the user’s home. The smart grid enables high system throughput as well as low energy consumption by allowing facilities to transfer the electricity from place to place in the system as economically and efficiently as possible. It also lets business and homeowners utilize electricity economically. Sustaining efficiency in communication is one of the major challenges in this application.

**Figure 10:**
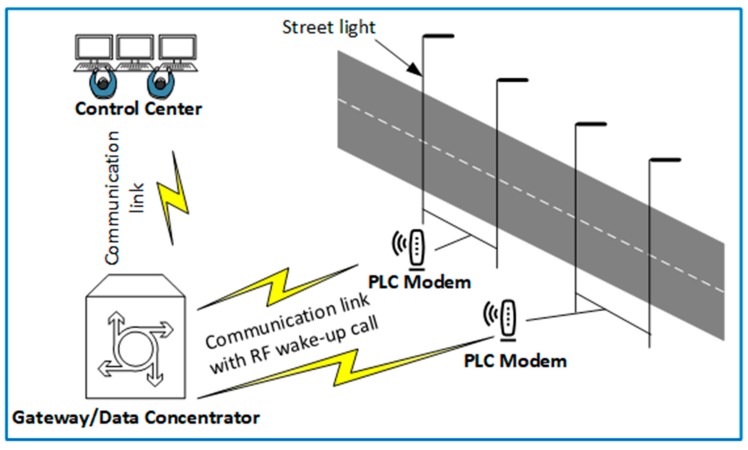
Description of Passive wake-up radio for smart grid application.

Power line communication (PLC) is a method by which smart grids achieve efficient point to point communication. However, the energy efficiency of this method is reduced in periodic communication scenarios such as smart street lighting. Passive wake-up protocols proposed in [118,119] eliminate this energy inefficiency by removing idle listening when the devices are on stand-by. Figure 10 shows a graphical representation of smart grid application in passive street light PLC. The PLC modem is attached to the passive wake-up radio, which allows the modem and the connected load (in this case street lights) to remain in a deep sleep and only wakes up after receiving a signal from the control center through the gateway. Data aggregation in smart grids is either on demand or periodic and hence, multi-hop capability, reduced latency, and high energy efficiency should be prioritized in the protocol design [120,121]. Protocols such as those proposed in [108] are suitable for the smart grid application.

### 4.4. Asset Tracking

Asset tracking in WSN is the employment of mobile wireless sensors on each of the assets to enable asset managers to track asset location and to indirectly monitor the asset usage status in real time. For cargo dispatching companies, an essential aspect of their business is the real-time status of their active assets. While some companies still use the manual tracking which is unreliable with higher overhead cost, the majority employs the smart asset tracking method. The RFID is most often used for this purpose with technologies, such as the ZigBee IEEE 802.15.4, adopting and improving upon the RFID functionality to enhance the asset tracking capabilities. RFIDs in the form of wake-up radios are used to track the products by their ID and the main radio handles the communication of other main information.

**Figure 11:**
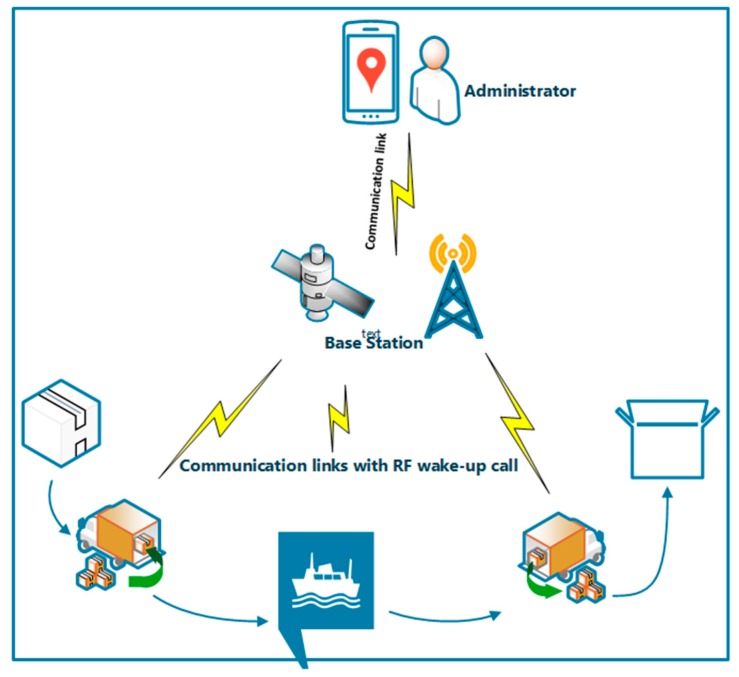
Description of passive wake-up radio for asset tracking application.

Asset tracking application specific quasi-passive wake-up radios are proposed in [122]. The wake-up radio design requirement for asset tracking should be able to operate for the long-term with bidirectional communication and high energy efficiency. Asset tracking system using the passive wake-up radio is shown in Figure 11. Although communication between two passive wake-up radios is considered for shorter range due to their low relatively low transmission power and receiver sensitivity. However, in Figure 11 the communication is initiated by a high transmission power base station, which can start the wake-up radio even at longer range. After the main radio is triggered to start-up by the wake-up radio, the main radio can send its information back to the base station over the long distance with high transmission power since it is active. The passive wake-up radio protocols proposed in [49,63,64] are suitable candidates for use in this application because of their low maintenance cost and long-term operation capability.

## 5. Research Challenges and Future Directions

In this section, we discuss the open research issues and practical challenges in the passive wake-up radio. While we mentioned earlier that most of the reported systems and protocols are evaluated based on theoretical analysis and simulation, we recommend moving forward with the development of testbed prototypes to accelerate the real-world implementation of wake-up radio technology. We also discuss the future research direction that can be engaged to solve these challenges.

### 5.1. Communication Range

A passive wake-up radio is designed based on passive circuits [64], which harvests energy from the incoming RF signal in the wake-up message to turn itself on and wake-up the main radio. This result in high energy efficiency since idle listening is eliminated and the wake-up radio does not need to be powered by a battery. However, the RF energy transmission in the form of the wake-up signal has a limited range, which is a major challenge of the system. The communication range of the passive wake-up radios is about 3 m [64], which is less than the active ones. Active wake-up radio typically achieves ~200 m in range [123]. The wake-up range is increased to almost 7.5 m in another protocol [103] with ultralow power radio triggered circuit, which is still not long enough as the normal active range. To achieve a design with longer range, the wake-up transmitter needs to send the signal at high power values. Improving the receiver sensitivity also increases the wake-up range. Unfortunately, both these approaches will increase power consumption. An RFID-based passive WuR technique is proposed in [82] for ultralong range communication, which is able to achieve a range of 22 m, although it is not fully passive design. This long range is achieved by a special design architecture, which composed of MEMSIC Iris mote, an augmented UHF RFID tag, and UHF RFID energy harvester.

### 5.2. Delay

Another major challenge of the passive wake-up radio is the increased latency. This is as a result of the limited RF energy from the sources, from which the receiver needs to harvest enough energy to power its circuitry. The increased delay in the trigger process and how to reduce it is still a significant issue in the research of passive schemes. The recent works which made an effort to limit the delay are mostly based on ultralow power circuitries such as [124], which are, however, not completely passive. The low latency in this architecture is achieved by making the propagation delay as the main criteria for selecting the comparator circuit elements. The reference voltage source of the comparator is optimized to eliminate the high delay due to long preamble needed in other designs.

### 5.3. System Integration and Standardization

The cost of designing and maintaining dual radio for the sensor node and the passive wake-up radio is a major challenge of this scheme. Instead of the current state of keeping detached components which is expensive, the components should be integrated together with the sensor platform intended for a specific application. Other aspects such as in-band or out of band, and addressing should also be applied on the same chip with main radio. Moreover, an integrated networking and system architecture is required for passive wake-up radios so that applications can be easily built upon it. The design of wake-up radios is yet to be standardized in terms of wake-up signal format, usable channels, frequency usage etc. An attempt is made in [125] for standardization in the 802.11 working group to have these design features standardized. By incorporating the passive WuR into the current electronic and hardware standards, it allows them to fully utilize RF energy harvesting, thus eliminating idle listening.

### 5.4. Contention and Channel Access

Contention and channel access are key issues in the design of passive wake-up radios. These issues are more prevalent when the main and wake-up radios need to share channels, and because they have dissimilar communication range, an asymmetric network is formed. This creates the need to develop protocols which are more responsive to channel variations. One way to resolve this is the use of cognitive radios with passive wake-up radios. The cognitive radios are recently being used in sensor networks as shown in [10,126]. They are not susceptible to collision and interference as against the regular radios, which adopt fixed channel allocation. The cognitive radios are able to achieve this because they can adaptively select and use the vacant spectrums in both licensed and unlicensed band. Using cognitive radio with passive wake-up radios will improve the overall performance of the system, especially in dense networks by reducing the energy usage and eliminating packet loss and collusion. Another solution to this challenge is the use of a Wi-Fi unit alongside wake-up radio so that transmission channels are selected dynamically as proposed in [127].

### 5.5. Application Specific Transmission Protocols

Application specific and adaptive protocols need to be developed for passive wake-up radios. This is necessary because different applications have dissimilar requirements. Similarly, passive wake-up radios are deployed in various physical environmental settings. Some of these applications have their sensor nodes in harsh environmental conditions, such as smart farm, structural monitoring, etc. Therefore, developing application adaptive and robust protocols is the best way to mitigate the high level of failure that these nodes are prone to and to improve fault tolerance in the system. For example, attempts are being made to develop passive wake-up radio adaptive protocols for medical application in [100,101,102]. These protocols mainly aim at improving energy efficiency as well as response time in order to achieve the medical application situations, which are, periodic/normal communication as well as random/emergency communication.

### 5.6. Mobility

Most of the schemes utilized for passive wake-up radios assume all the sensor nodes to be stationary. However, mobility is needed in some IoT applications, such as large and sparsely deployed sensor nodes environments, where data aggregation by a central node may be challenging. Some of the passive WuR schemes are adapting to the mobility environment while attempt to improve energy efficiency, such as [79], which uses a mobile sink for an initial wake-up in the system. For example, a mobile car, working as a base station, drives along the road to wake-up each node and collect data. Therefore, there is an opportunity to develop robust protocols that can adapt to the rapid topology change in mobile networks.

## 6. Conclusions

This paper reviews the RF passive wake-up radio concept from hardware to the MAC layer design. Increasing attention has been given to traversing the different aspects of the passive wake-up radio design setting. We provided a comprehensive and systematic classification of the current state-of-the-art techniques available in the literature. From the literature review, it can be observed that there has been huge progress in the development of passive wake-up radio hardware. The associated performance parameters, such as sensitivity, range and power dissipation is evaluated and compared. The growing number of communication protocols that are utilized by the passive WuR features have also been analyzed.

The study shows that RFID based wake-up radios are generally superior to other technologies based on the relative higher sensitivity (hence, offer high wake-up range), lower V_in_-to-V_out_ ratios, implying that lower turn-on input voltage can produce a higher output voltage and hence, higher efficiency.

The MAC protocols are also having issues of contention, channel access and delays caused by the time required to harvest enough energy to power it up. The key evolving application areas where the passive wake-up radios are utilized have been discussed, such as environmental monitoring, medical, smart grid and asset tracking. Open research issues and practical challenges in the passive wake-up radio have been highlighted, which include communication range, delay, channel access, application-specific protocols and mobility. In addition, several solutions and research opportunities have been highlighted to solve these challenges in order to reduce the technological barrier towards the realization of passive wake-up radio technology. Furthermore, most of the passive WuR MAC protocols evaluation is based on theoretical analysis and simulation, which can be further verified in the testbed prototypes and real implementation by researchers in the future. Our work has described the great potential of passive wake-up radios to improve the energy efficiency in wireless sensor networks in IoT applications.

## Figures and Tables

**Table 1:** Passive wake-up radio technology and its performance characteristics and properties.

Addressing Type	Components Used	Reference Example	Freq. (MHz)	Min. V_in_ (V)	V_out_ (V)	Sensitivity (dBm)	Data Rate (kbps)	Power Dissipated (µW)	Implementation
ID-Based	Discrete	[72,73]	433	-	0.6	−23	-	84	Simulation
		[74]	2400	0.05	1.13	−28	1	39	Prototype
	RFID	[75]	915	-	>1.9	−4	-	-	PCB
		[76]	-	-		-	-	100	Prototype
		[21]	915	0.92	1.8	−80	1 packet/min	0.043 (mJ/pkt)	Prototype
		[77]	900	0.92	1.8	−80	1 packet/min	0.04 (3 mJ/pkt)	Prototype
		[81]	915	0.15	2.074	−20	-	0.25	PCB
		[79]	900	-	-	−86	-	-	Prototype
		[80]	915	0.15	1.5	−20	-	-	PCB
				0.34					
		[82]	868	1.8	-	N/A	-	54	Prototype
	130-nm CMOS	[83]	2400	1	-	−47	200	2.3	Prototype
	65 nm CMOS	[84]	2400	-	0.03	−56.5	8.192	0.236	Prototype
						−39		0.104	
	65 nm CMOS	[89]	2400	1	-	−50	100	13	Simulation
Broadcast	180 nm CMOS	[90]	900	0.8	0.7	−17	-	2.64	Prototype
	130 nm CMOS	[91]	868	-	0.8	−33	100	0.5	Simulation
	130 nm CMOS	[92]	915	1.2	-	−53	100	0.2	Prototype
	180 nm CMOS	[93]	2400	1.2	1.8	−23	-	5	Simulation

**Table 2:** Key features and characteristics of the MAC protocols for the wake-up radio.

Network	Implementation	Ref.	Key Innovation	Downside	Channels	Wake-Up Signal	Collision Avoidance
Single Hop	Simulation	[99]	Dynamic channel allocation & narrowband channel for RTS/CTS	Channel allocation overhead	Multiple	Signal with ID & ACK	RTS/CTS, distributed channel Assignment and CCA
		[100,101]	TDMA scheme for multiple access and resource allocation	-	Out-of-band	Beacon with ID & ACK	Wake-up schedule table
		[102]	Introduced the piggyback approach on wake-up and ACK messages	-	Out-of-band	Beacon with ID	Unique slot allocation, unique frequency for sending a wake-up packet.
		[104]	Wake-up radio that supports mobility	-	Out-of-Band	Beacon with ID	CSMA/CA
		[105]	Combining WuR with energy harvesting	More time needed to harvest enough energy causing delay	In-band	Beacon with ID & ACK	CCA
		[107]	Combining WuR with energy harvesting	More time needed to harvest enough energy causing delay	Out-of-Band	Signal with ID	Unique slot allocation for each node
		[113]	Joint downlink/uplink RF wake-up (receive the WuS in DL and transmit data in UL)	Increased false wake-up rate	Out-of-Band	Signal with ID	Unique frequency for sending a wake-up packet.
		[114]	Transfer of wake-up data and power for wireless-powered wake-up receiver WPWRx by utilizing time switching.	Repeated preamble needs to be received before determining the exact WuS arrival time	Out-of-Band	Preamble	TDMA
	Test-bed	[112]	Nodes utilize three channels receiver which generates a wake-up signal upon detection of a data signal.	Delay due to time for MCU to wake-up from sleep.	Out-of-Band	Signal with ID	NA
Multi Hop	Simulation	[108]	RTS/CTS mechanism used to check the problem of hidden terminal overhearing.	Uses the same channel for wake-up preamble and signal	In-band	Preamble	CSMA/CA and RTS/CTS exchange
	Simulation	[109]	Nodes only wake-up for each packet and then go back to sleep	Energy waste due to long idle listening & channel reservation	Out-of-band	Signal with ID	CCA, binary exponential back-off mechanism
	Simulation	[110]	Employs predistribution pairwise key management protocol for security	Delay due to the warm-up time required to wake-up	Multiple	Signal with ID	Unique frequency for sending a wake-up packet
	Simulation	[111]	Redundant residue number systems (RRNS) ECC is used to improve reliability and reduce retransmission.	Delay due to ECC overhead in coding and decoding of packets.	-	-	TDMA
	Test-bed	[79]	Introduced mobile sink, which moves around to wake-up other nodes.	All nodes within the wake-up range wake-up	Out-of-Band	Signal only	NA
